# Single-cell RNA sequencing reveals distinct immunology profiles in human keloid

**DOI:** 10.3389/fimmu.2022.940645

**Published:** 2022-08-03

**Authors:** Cheng Feng, Mengjie Shan, Yijun Xia, Zhi Zheng, Kai He, Yingxin Wei, Kexin Song, Tian Meng, Hao Liu, Yan Hao, Zhengyun Liang, Youbin Wang, Yongsheng Huang

**Affiliations:** ^1^ Department of Plastic Surgery, Peking Union Medical College Hospital, Beijing, China; ^2^ Department of Plastic Surgery, Peking Union Medical College Hospital, Chinese Academy of Medical Sciences, Peking Union Medical College, Beijing, China; ^3^ Institute of Basic Medical Sciences and School of Basic Medicine, Chinese Academy of Medical Sciences and Peking Union Medical College, Beijing, China; ^4^ Key Laboratory of Conservation and Application in Biodiversity of South China, School of Life Sciences, Guangzhou University, Guangzhou, China; ^5^ Department of General Surgery, Peking Union Medical College Hospital, Chinese Academy of Medical Science and Peking Union Medical College, Beijing, China

**Keywords:** keloid, single-cell RNA sequencing, fibroblast, macrophage, immunology profiles

## Abstract

Keloids, characterized by skin fibrosis and excessive accumulation of extracellular matrix, remain a therapeutic challenge. In this study, we systematically capture the cellular composition of keloids by the single-cell RNA sequencing technique. Our results indicated that there are significant differences in most cell types present between 12 pairs of keloid and adjacent normal tissue. We found that fibroblasts, endothelial cells, mast cells, mural cells, and Schwann cells increased significantly in keloid. The proportion of mesenchymal fibroblast subpopulations in keloids was markedly higher than those in the surrounding normal skin tissue. Furthermore, we found that the immune profiles between two groups varied significantly. The proportion of macrophages in the keloid was significantly elevated compared to the surrounding normal tissue, while cDC2 cells significantly decreased. Hotspot and pseudotime trajectory analysis indicated two modules of macrophage cells (Module2: highly expresses RNASE1, C1QA, CD163, CD14, C1QC, FCGRT, MS4A7; Module10: highly expresses APOC1, CTSB, CTSL, TYROBP), which exhibited the characteristics of tumor-associated macrophages, were upregulated in more-advanced keloid cells. Subsequently, the analysis of cellular communication networks suggested that a macrophage-centered communication regulatory network may exist in keloids and that fibroblasts in keloids may facilitate the transition and proliferation of M2 macrophages, which contributes to further comprehension of the immunological features of keloids. Overall, we delineate the immunology landscape of keloids and present new insights into the mechanisms involved in its formation in this study.

## Introduction

Keloid (K) is the result of abnormal tissue repair caused by a variety of factors (1, 2). Because of its clinical characteristics of continuous growth, no self-limitation, easy recurrence after resection, and invasive growth to normal skin, it has brought serious damage and psychological burden to patients (3). Keloid tissue is composed of cellular components and extracellular matrix components (ECMs) (4, 5). The immune cells play a decisive role in the biological behavior of keloids. Keloid fibroblasts proliferate abnormally and secrete too much extracellular matrix, which have a strong proliferation ability (6). Keloid fibroblasts have always been considered to be the cause of the vigorous proliferation of keloids, but the influence of the surrounding immune microenvironment has been ignored. Keloid fibroblasts and immune cells interact with each other and develop synergistically and the complex regulation of their molecules constitutes the immune microenvironment of keloids (4). Studying the changes of the immune microenvironment and its regulatory mechanism will benefit keloid treatment by regulating important immune cells.

Single-cell transcriptome sequencing is a novel technique for sequencing the transcriptome of a single cell as a specific research object and performing bioinformatics statistical analysis on the obtained data (7). Based on technologies such as microfluidics and barcode labeling, a huge number of large cells can be rapidly and efficiently labeled, sequenced, and analyzed, and gene expression profiles and differences at the single-cell level can be obtained (7, 8). Several previous studies have applied single-cell sequencing techniques in the field of keloids, but they mainly focus on the study of extracellular matrix and fibroblasts (9–11). This study focuses on the study of the immune microenvironment of keloids, revealing immunology profiles in human keloids.

## Methods

### Patient

This study was approved by the Medical Ethics Committee of Peking Union Medical College Hospital (JS-2907). All participants provided written informed consent. From October 2019 to January 2022, 12 patients with keloids were included in this study. Surgically excised skin tissue was collected from the normal skin tissue of cosmetic surgery patients. Keloid skin (group K) and the surrounding normal skin (group N) served as controls for single-cell sequencing.

### Tissue dissociation and preparation

Fresh keloid and surrounding normal skin tissue was obtained and stored in GEXSCOPETM Tissue Preservation Solution and transferred to the laboratory frozen immediately. All specimens were cleansed thrice with Hanks Balanced Salt Solution (HBSS) and prepared into 1–2-mm pieces, which were subsequently digested in a GEXSCOPETM tissue dissociation solution (Singleron Bio Com, Nanjing, China). After the samples were filtered and centrifuged and the supernatant discarded, the sediment was resuspended in phosphate buffered saline (PBS) (HyClone, China). Specimens with excess erythrocytes were treated with GEXSCOPETM Erythrocyte Lysate (Singleron Bio Com, Nanjing, China) for de-erythrocyte removal. Samples were stained with trypan blue (Sigma, United States) and evaluated microscopically.

### Library construction and single-cell sequencing

The cell concentration of the single-cell suspension was maintained at 1 × 10^5^ cells/ml and loaded onto a microfluidic chip. Single-cell RNA-seq (scRNA-seq) libraries were constructed using the GEXSCOPETM Single Cell RNA Library Kit (Singleron Biotechnologies) (12). Individual libraries were diluted to 4 nM and sequencing was completed on the Illumina HiSeq X sequencing platform, selecting the 150-bp double-end mode.

### Contamination removal

The decontx algorithm in the celda package (v.1.3.8) performed environmental RNA removal using raw UMI counts and cell clusters calculated by the FindClusters function. A default random seed was applied throughout the analysis to ensure reproducibility.

### scRNA-seq quantifications

Raw data, which were processed after filtering out reads1 without poly T, screening out reads2 (fastp V1) with connectors and poly A tails, and comparing with the reference genome from the ensembl database (STAR 2.5.3a and featureCounts 1.6.2), were generated as a gene expression matrix (13). The data were grouped according to cell barcodes, and the number of UMIs was calculated per gene in each cell. With the Seurat package (http://satijalab.org/seurat/, R package, v.3.0.1), gene expression matrices were created as Seurat objects to perform cell type annotation and downscaling clustering analysis (14). The FindCluster function was set to a resolution of 0.6 to cluster the mixed cells, and the FindMarker function was applied to identify differentially expressed genes in different clusters of cells. The functional annotation of gene sets was implemented with the clusterProfiler package (15).

### Quality control, dimension-reduction, and clustering (Scanpy)

For each sample dataset, the expression matrix was filtered through the Scanpy package v1.8.2 (16) based on the following exclusion criteria: 1) cells with the maximum 2% of UMI counts; 2) cells with the maximum 2% of gene counts; 3) cells with fewer than 200 genes; 4) genes expressed in less than five cells; and 5) cells with >50% mitochondrial gene content. For the screened raw count matrix, normalization was performed by dividing by the total counts per cell and calculating the natural logarithm. The first 2,000 variable genes were selected, and the matrix was subjected to principal component analysis, with the first 20 principal components serving for subsequent clustering and dimensionality reduction analysis. The removal of batch effects between samples was implemented by Harmony v1.0 (17). The clustering of cells was achieved by the Louvain algorithm, setting the resolution parameter to 1.2, and visualized by Uniform Manifold Approximation and Projection (UMAP).

### Differentially expressed gene analysis

Differentially expressed genes were identified by the FindMarker function based on the Wilcox likelihood ratio test with the qualification that they are expressed in more than 10% of cells in one cluster and with an average log (fold change) greater than 0.25.

### Cell type annotation

The cell types of each cluster were determined by the expression of the identified differential genes and typical cellular markers reported in the previous literature, and the visualization of typical differential genes in cell clusters was achieved using heatmaps.

### Pathway enrichment analysis

Gene ontology (GO), Kyoto Encyclopedia of Genes and Genomes (KEGG) analysis, and GSVA pathway enrichment analysis were used to predict the biological function, cellular composition, and possible pathways involved in differentially expressed genes, with an adjusted P-value of less than 0.05 being considered statistically significant (18).

### Trajectory analysis

Monocle2 was employed for pseudo-temporal trajectory analysis, and the DDRTree method was adopted for dimensionality reduction to map the differentiation of macrophage subtypes in keloid. The results were visualized by the “plot_cell_trajectory” function (19).

### RNA velocity

The velocyto package was utilized to analyze BAM files containing target cells and genomic GRCh38 (hg38) for RNA rate analysis, and the results were projected onto UMAP plots for Seurat clustering analysis for visual consistency (20, 21).

### Single-cell entropy analysis

SLICE (version 0.99.0) was used to evaluate the stemness of cells by the entropy of gene expression based on single-cell expression profiles. After removing and ribosomal genes, a SLICE object was created to perform a bootstrap calculation of single-cell gene entropy values by the getEntropy function.

### Cell–cell interaction analysis (CellPhoneDB)

Cellphone DB v2.1.0 was equipped with a detailed receptor–ligand database to predict cell–cell interactions to identify cell pairs in the presence of reciprocal regulation or communication. The number of ineffective distributions of reciprocal exchanges was set to 1,000. The predicted interaction pairs with a p-value <0.05 and of average log expression >0.1 were considered as significant (22).

### Transcription factor regulatory network analysis (scenic)

The SCENIC R toolkit was used to construct transcription factor networks on scRNA expression matrices. The GENIE3 package inferred coexpression modules between transcription factors and candidate target genes. The RcisTarget package performed cis-regulatory motif analysis for each coexpression module, constituting a gene regulatory network module containing both TF and target genes. The AUCell package calculated the AUC values of this genome to assess the activation of the gene regulatory network modules in cells.

### Expression program analysis

The transcriptional programs were extracted by the cNMF algorithm, the top 50 genes were taken as the meta-signature, and the score of each program per cell was computed by the meta-signature. The meta-program was calculated and there was hierarchical clustering according to the Pearson correlation between each program.

### Immunohistochemical staining

Immunohistochemical experiments were performed according to the streptavidin–biotin–peroxidase complex procedure. The tissue sections of keloid and surrounding skin were dewaxed and rehydrated with xylene and different concentrations of ethanol, kept in 3% hydrogen peroxide solution for 20 min, and subsequently rinsed with distilled water for 5 min. Samples were treated with primary antibody working solutions against CD8 (ProteinTech, #66868-1-Ig,1:400), CD4 (ProteinTech, #67786-1-Ig, 1:450), CD68 (ProteinTech, #66231-2-Ig,1:1,000), iNOS (ProteinTech, #22226-1-AP,1:500), IL-10 (ProteinTech, #60629-2-Ig,1:500), and IL-12 (Abcam, #ab9992, 1:1,000), respectively. The samples were incubated with biotin-labeled secondary antibodies for 30 min at 37°C. The sections were added with an appropriate amount of an alkaline phosphatase–labeled streptavidin working solution and incubated at 37°C for 30 min. After rinsing with PBS, the chromogenic agent was treated for 15 min. The slices were restained, dehydrated, and sealed.

## Results

### Characteristics of the overall cell types in human keloids by single-cell RNA-seq

**Figure 1:**
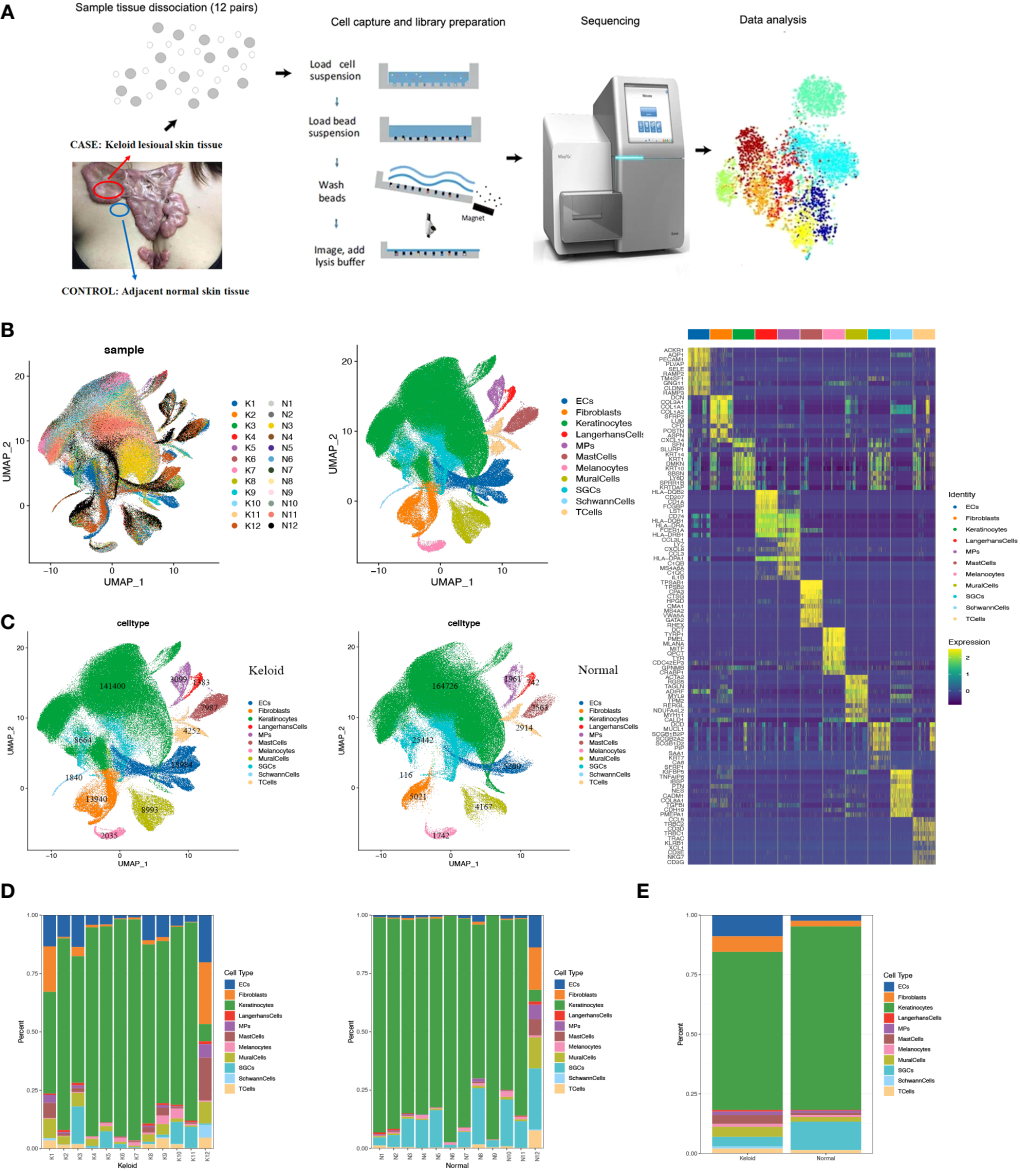
Single-cell RNA-seq (scRNA-seq) reveals the cellular diversity and heterogeneity of keloid skin tissue. **(A)** Schematic representation of the experimental procedure. Keloids and adjacent normal skin tissues were harvested separately at the time of surgery (n=24). **(B)** Uniform Manifold Approximation and Projection (UMAP) of unbiased clustering of 427,171 cells reveals 11 cellular clusters. Clusters are distinguished by different colors. The number in the parenthesis indicates the cell count. **(C)** Hierarchical clustering of the clusters based on the average expression of the 2,000 most variable genes. **(D)** Comparison of the ratio of cells in keloids and adjacent normal skin tissues. **(E)** Representative molecular signatures for each cell cluster. SGCs, sweat gland cells; ECs, vascular endothelial cells; MPs, mononuclear phagocytes.

To explore the immunological profile of keloids and to determine cellular heterogeneity, we used single-cell RNA sequencing to compare keloid skin tissue (CASE) with matched adjacent normal tissue (CTRL) from 12 patients (**Supplementary Table 1**). The 24 samples were dissociated into single cells and subjected to single-cell RNA-seq (**Figure 1A**). The transcriptomes of 427171 number cells (CASE: 213105; CTRL: 214066) were yielded after quality control. Based on hierarchical clustering and established lineage-specific marker genes (**Supplementary Table 2**), we assigned these clusters to 11 cell lineages (**Figures 1B–D**). The keratinocyte lineage (marked by KRT1, KRT10, KRT14, KRT5) (23, 24) constituted the maximum percentage (71.7%) of cells. Endothelial cells (ECs) (marked by PECAM1, CDH5, VWF, and KDR) (25) represented the second biggest proportion (5.7%) of cells, while the fibroblast lineage made up the third biggest proportion (4.4%) of cells (marked by DCN, COL1A1, and COL1A2) (25, 26). Additionally, other classic skin lineages were identified, like sweat gland cells (labeled by DCD and AQP5) (24), mural cells (marked by RGS5, ACTA2, TAGLN, and MYLK) (26, 27), Langerhans cells (marked by CD207 and CD1A) (27), melanocytes (marked by MLANA, PMEL, DCT, and TYRP1) (28), and Schwann cells (marked by S100B, PMP22, and PLP1) (29, 30). These clusters revealed diverse molecular profiles (**Figure 1E**), capturing the cellular variety and heterogeneity of keloid tissue.

### Characteristics of fibroblasts in keloids

**Figure 2:**
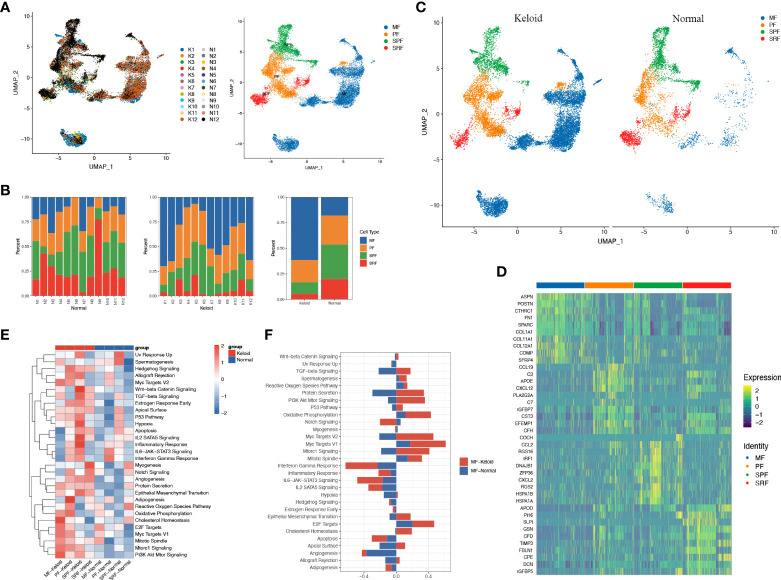
Fibroblasts of keloid and normal skin tissue subcluster into distinct cell populations. **(A)** Subclustering of keloid and normal tissue fibroblasts identified four distinct subtypes: mesenchymal (MF), pro-inflammatory (PF), secretory-papillary (SPF), and secretory-reticular (SRF). **(B)** Demonstration of the ratio of the four fibroblast subpopulations in keloids and adjacent normal tissue. **(C)** Color-coded UMAP plot is shown, and each fibroblast subcluster is defined. **(D)** Heatmap plots showing representative differentially expressed genes between the four fibroblasts in the keloids. **(E)** Gene ontology (GO) biological process enrichment analysis of differentially expressed genes in the four fibroblasts between keloid and normal scars. **(F)** GO enrichment analysis of differentially expressed genes in mesenchymal fibroblasts.

To account for the significant changes in fibroblasts during the fibrotic process in keloids (**Figures 1C, D**) and the dominance of fibroblasts in the pathogenesis of fibrosis, we proceeded to perform an unsupervised clustering analysis of fibroblasts in all keloids and normal skin. A previous study proved that normal human dermal fibroblasts can be divided into four subpopulations: secretory-papillary (SPF), secretory-reticular (SRF), mesenchymal (MF), and pro-inflammatory (PF) (31). Hierarchical cluster analysis suggested that fibroblasts from keloid and normal tissue in our study could also be divided into these four subpopulations (**Figures 2A–C**) (**Supplementary Table 2**). Moreover, we found that the proportion of MF increased significantly in keloids compared to normal tissue, while SPF and SRF decreased (**Figure 2B**). We further explored differentially expressed genes between these subpopulations in keloid. We found that genes associated with matrix synthesis and fibroblast and osteoblast differentiation, including ASPN, POSTN, CTHRC1, FN1, and COL11A1, and secretory proteins, such as SPARC, SFRP4, COMP, COL11A1, and COL12A1, were enriched in the mesenchymal subpopulation (**Figure 2D**).

scRNA-seq analysis demonstrated that the ratio of keloid mesenchymal fibroblasts was markedly elevated in comparison to normal skin. Thus, our following work concentrated on this fibroblast subpopulation. The heatmap indicated a marked difference in gene expression between the mesenchymal subpopulation and other subpopulations (**Figure 2D**). The keloid and normal skin varied in the expression of a variety of genes among fibroblast subtypes, and this differentiation was particularly evident in mesenchymal fibroblasts. Genes involved in extracellular matrix formation including COL1A1, COL3A1, POSTN, COL1A2, FN1, and ASPN were significantly upregulated in keloid compared to the normal skins (**Supplementary Figure 1A**). GO analysis revealed that differentially expressed genes in the mesenchymal subpopulation were associated with protein secretion, oxidative phosphorylation, cholesterol homeostasis, MYC, and E2F (**Figures 2E, F**), whereas SPF was correlated with TGF-β, hypoxia, epithelial mesenchymal transition, angiogenesis, inflammatory response, apoptosis, and IL2 (**Figure 2E**). Furthermore, GO analysis indicate that angiogenesis, protein secretion, EMT, and the PI3K/AKT pathway enriched in all of the four types of keloid fibroblast (**Figure 2E**).

### Characteristics of immunology profiles in human keloids

**Figure 3:**
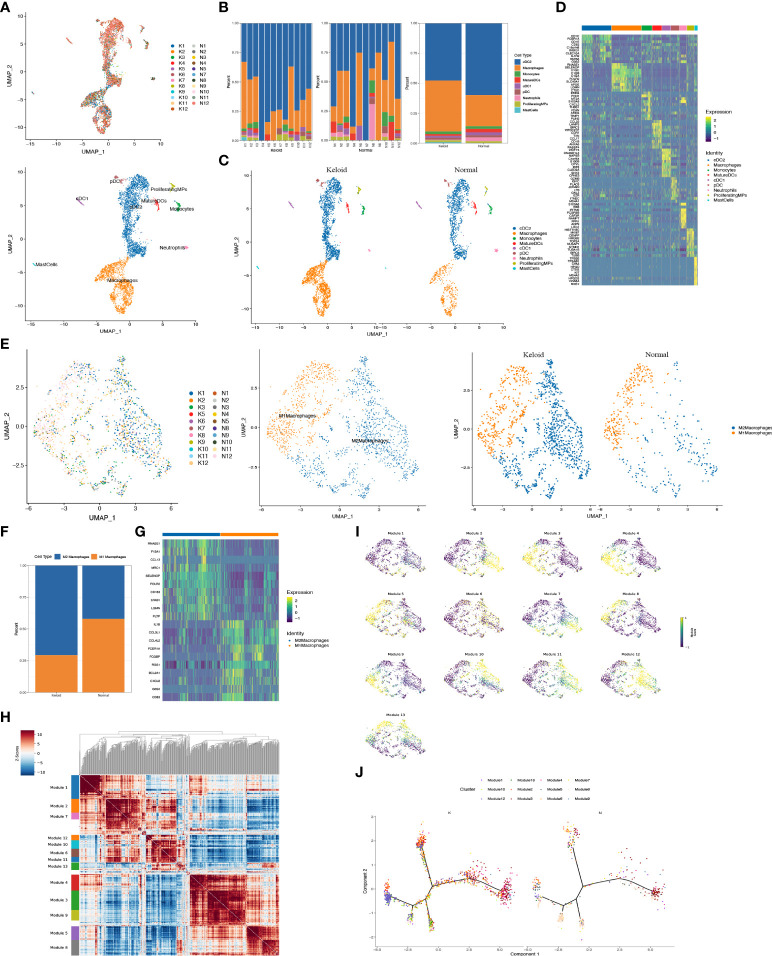
Characteristics of immunology profile in human keloids. **(A)** Color-coded UMAP plot is shown, and each immune cell subpopulation is defined as: macrophages, conventional type 1 dendritic cells (cDC1s), conventional type 2 dendritic cells (cDC2s), mature dendritic cells (MatureDCs), proliferating mononuclear phagocytes (ProliferatingMPs), plasmacytoid dendritic cells (pDCs), mast cells, neutrophils, and monocytes. **(B, C)** The proportions of immune cell subpopulations in keloid and adjacent normal tissue were shown. **(D)** Heatmap plots showing representative differentially expressed genes between the different types of immune cells in the keloids. **(E, F)** Demonstration of the ratio of M1 and M2 macrophage subpopulations in keloid and adjacent normal tissues. **(G)** Heatmap showing representative differentially expressed genes in M1 and M2 macrophage subpopulations. **(H, I)** Demonstration of representative modules of macrophages in keloid. **(J)** Pseudo-trajectory and cell source transition of macrophage cells between keloid and adjacent normal tissue.

The scRNA-seq analysis revealed that the immune profiles between the keloids and adjacent tissues also varied significantly, especially the mononuclear phagocytic system (MPS). The proportion of macrophages was considerably elevated in keloids compared to normal tissue, while the proportion of cDC2 dendritic cells was reduced (**Figures 3A–C**). The change of other myeloid cells type is small (**Figure 3B**). The heatmap indicated that gene expression was markedly diverse among MPS (**Figure 3D**), with increased genes in macrophages including some RNASE1, SELENOP, C1QC, C1QB, C1QA, FOLR2, SLC40A1, APOE, LGMN, and CTSD (**Figure 3D**). The increased genes in the cDC2 included CD1C, FCENA, CD1E, CD52, INSIG1, CLEC10A, IL1R2, HSPAa, and GOS2. Furthermore, it indicated that the ratio of M2 macrophage subpopulations was higher than M1 macrophage subpopulations in keloid (**Figures 3E, F**), and the heatmap showing representative differentially expressed genes in M1 and M2 macrophage subpopulations (**Figure 3G**). The expression of genes that participate in mitochondrial oxidative phosphorylation processes such as MT-CO1, MT-CYB, MT-ND5, MT-CO3, and genes involved in extracellular matrix binding and formation, like COL1A1 and SPARC, were elevated in M2 macrophages in keloids, while the expression of extracellular matrix catabolic proteins as MMP9 was diminished (**Supplementary Figure 1B**). In addition, the hotspot analysis of macrophage cells, in which cells are grouped according to the degree of similarity in their transcriptomes, shows 13 modules (**Figures 3H–J**). Among them, Module2 (highly express RNASE1, C1QA, CD163, CD14, C1QC, FCGRT, and MS4A7) and Module10 (highly express APOC1, CTSB, CTSL, and TYROBP) are more likely to display the characteristics of tumor-associated macrophages (TAMs).

**Figure 4:**
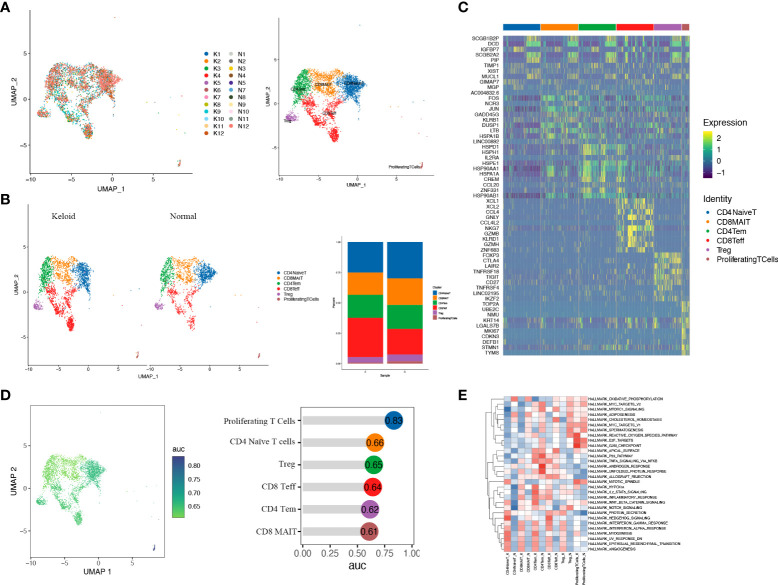
T cells of keloid and normal skin tissues were subdivided into different cell subsets. **(A)** Six different subsets of T cells are identified in keloid and normal tissues: CD4+ naïve T cells (CD4NaiveT), CD4+ effector memory T cells (CD4Tem), CD8+ effector T cells (CD8Teff), CD8+ mucosa-associated invariant T cells (CD8MAIT), CD4+ regulatory T cells (Treg), and proliferating T cells. **(B)** The ratio of six T-cell subsets in keloid and adjacent normal tissues is shown. **(C)** Heatmap illustrating representative differentially expressed genes among the six T-cell subsets. **(D)** Augur analysis indicates that proliferative T cells are the cell type with the greatest variation among subsets. **(E)** Enrichment analysis of differentially expressed genes in the six T-cell subpopulations.

**Figure 5:**
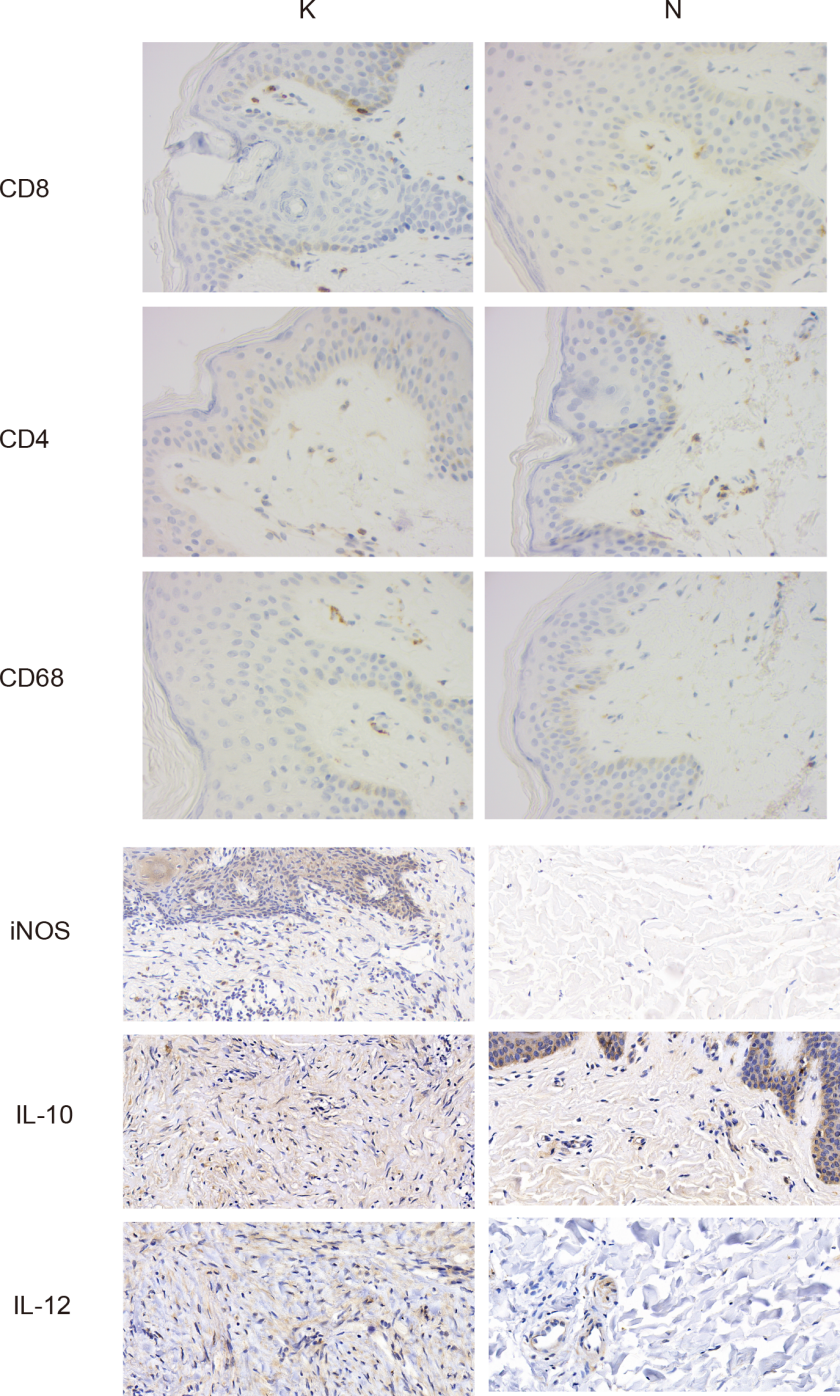
Typical images of immunohistochemical staining in the keloid (group K) and surrounding normal skin tissue (group N). Gross appearance: ×400.

Considering the essential role of T cells in immunity, we proceeded to perform a descending clustering of T-cell populations and identified six cell types, including CD4+naïve T cells, CD4+ effector memory T cells, CD8+ effector T cells, CD8+ mucosa–associated invariant T cells, CD4+ regulatory T cells, and proliferative T cells, with the proportion of CD8+ effector T cells predominating in keloids (**Figures 4A–C**). The ratio of CD4+naïve T cells and proliferating T cells was decreased in keloids compared with adjacent normal skin tissue (**Figure 4B**), and Augur analysis suggested that proliferative T cells were the cell type with the greatest variation (**Figure 4D**). Enrichment analysis suggested distinct differences in T cell subtypes between the two groups in terms of angiogenesis, myogenesis, interferon alpha response, and interferon gamma response (**Figure 4E**). The ratio of immune cells was verified by immunohistochemistry, and the results indicated that there were more CD8+ and CD68+ cells and fewer CD4+ cells in the keloid compared to the surrounding normal skin tissue (**Figure 5**). The expression of some inflammatory factors also differed between the two groups. The expression of iNOS, IL-10, and IL-12 were higher in keloids (**Figure 5**). The results of the image mass spectrometry of an extensive immune panel including 25 markers indicated that CD68, CD14, and CD16 positive macrophages and CD11c-positive DC cells were enriched in keloids, which is consistent with the results of scRNA-seq (**Supplementary Figure 2**).

### Cell communications

**Figure 6:**
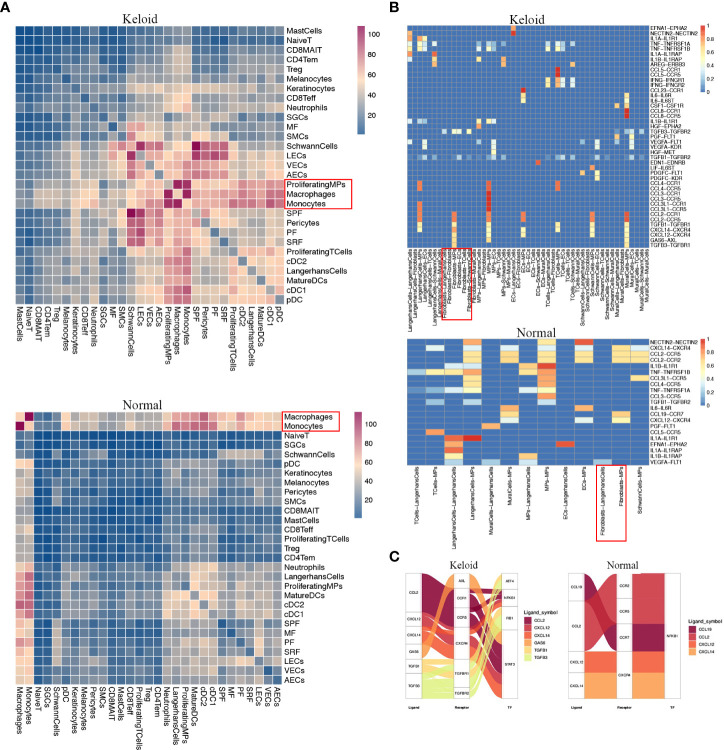
Potential ligand–receptor interactions analysis in fibroblast subpopulations. **(A)** Heatmap showing the numbers of interpopulation communications with each other in keloid and adjacent normal tissue. **(B)** The ligand–receptor pairs exhibit significant changes in specificity between any one of the populations and any one type of fibroblast in adjacent normal tissue versus keloid. **(C)** Putative and relative signaling or transcriptional factors (TFs) within fibroblasts and other cell populations’ keloid and adjacent normal tissue.

We investigated cell–cell communication with CellPhoneDB, and the results demonstrated different types of cellular communication between keloids and adjacent normal tissues. An intensive communication network between fibroblasts and other cells was observed in normal skin tissue and keloid (**Figures 6A, B**). The most abundant interactions in both cases appeared between the four fibroblast subpopulations, indicating the importance of fibroblast interaction signals in the dermis. Furthermore, we present the observation of a dense communication network between macrophages, DC cells, and other monocytes, which also indicated the importance of these cells in skin. In addition, we found that the cell interactions among keloids are much more strong than normal tissues.

Since macrophages may be the major immune cells contributing to keloids, we evaluated specific interactions among macrophages and additional cell types (**Figure 6A**). The interaction of macrophages with fibroblasts was strikingly noted (**Figure 6A**). Fibroblasts in keloids may specifically interact with macrophages and other MPs *via* TGFB2–TGFBR2, TGFB3– TGFBR3, CCL2-CCR1, and CCL2-CCR5 binding (**Figure 6B**), which play inhibitory roles on the target cells. We also found that the interactions between macrophage cells with the T cell and macrophage itself were remarkable in keloid. Furthermore, we found that the fibroblasts may activate the transcription factors ATF4 and RB1 in MPs (**Figure 6C**). Since the downstream genes of RB1 are related with M2 macrophages, for example: C1QC,C1QB,MRC1, fibroblasts in keloid may be beneficial for the transition and proliferation of M2 macrophages.

## Discussion

Keloid scars are the consequence of an overexpansion of the extracellular matrix during wound healing. Multiple factors, consisting of limited autophagy-mediated degeneration, the chronic inflammatory status, and the reorientation of fibroblasts into myofibroblasts, contribute to excessive skin fibrosis (32–34). A multitude of immune cells and cytokines are involved in the formation and progression of the above pathological processes. Despite extensive research on keloids, the key mechanisms of keloids have not been thoroughly elucidated, especially the role of immune cells including macrophage cells, dendritic cells, and T cells in keloid pathology is poorly understood, and methods for early identification, prevention, and treatment of keloids are still scarce (35).

With the increasing maturity of high-throughput sequencing technology, transcriptome sequencing has been widely adopted in the fields of biological physiological mechanism research, genetic breeding, and pathological analysis. Traditional bulk RNA-seq can merely reflect the gene expression of a vast collection of cells, while for some tissues with high cell heterogeneity, bulk RNA-seq does not fully reveal the fine-scale cellular distribution, and it is essential to investigate the mechanisms of gene expression variations and cellular differentiation at the level of single cells (36). Therefore, the aim of this paper is to depict the cellular landscape of keloids and highlight the types of immune cell composition in keloids. These findings facilitate a better understanding of the immune microenvironment of keloids, which may provide new targets for the immunotherapy of keloids.

Keloids are composed of abundant, swirling, irregular collagen fibers that are partially infiltrated with inflammatory cells and chemokines, forming their unique histological structure. Fibroblasts are recognized as the central cells of keloids, but the immune infiltrative profile around keloids remains underevaluated (37). This study revealed a significant increase in the proportion of mesenchymal fibroblasts in keloids, which are involved in the expression of genes related to matrix synthesis and fibroblast and osteoblast differentiation. The identification of the cell subpopulations of mononuclear phagocyte clusters implied a decreased proportion of cDC2 in keloids compared to normal tissues. As key antigen-presenting cells that initiate and shape the immune response, dendritic cells are critical in recognizing pathogens, promoting naïve T-cell activation, and maintaining peripheral T-cell homeostasis (38, 39). A recent study by Mayer et al. revealed that interleukin (IL)-13, produced by type 2 innate lymphocytes, is an essential molecule for maintaining cDC2 homeostasis and suppressing inappropriate inflammatory responses through the IL-13-STAT6 axis (40). IL13 downregulates the macrophage activity and inhibits the production of pro-inflammatory cytokines and chemokines (41, 42). Thus, the reduction of cDC2 in keloids may be associated with the downregulation of IL-13 expression, and the possible inappropriate inflammatory response associated with the downregulation of IL-13 expression is consistent with the pathogenesis of keloids.

Macrophages can polarize into M1-type and M2-type macrophages under different microenvironments in addition to their original ability to phagocytose bacteria and antigen presentation, and M2-type macrophages are critical for the induction of skin fibrosis during wound healing (43). In our study, it indicated that the ratio of M2 macrophage subpopulations was higher than M1 macrophage subpopulations in keloid. Furthermore, two modules of macrophages (Module2: highly expresses RNASE1, C1QA, CD163, CD14, C1QC, FCGRT, and MS4A7; Module10: highly expresses APOC1, CTSB, CTSL, and TYROBP), which exhibited the characteristics of TAMs, increased significantly in keloid. Since TAMs usually refer to the versatile macrophage M2 phenotype, M2 cells perform their multiple tasks by interacting with Th2 cells, regulatory T cells (Tregs), and fibroblasts (44, 45). Their ability to scavenge receptors, promote angiogenesis, and express anti-inflammatory cytokines is superior to that of M1-type macrophages (45, 46). M2 macrophages in fibrotic diseases maintain fibroblast proliferation, enhance the expression of the tissue inhibitors of metalloproteinases, induce fibroblast differentiation into myofibroblasts by secreting growth factors like the transforming growth factor (TGF-β1) and platelet- derived growth factor (PDGF), and thus directly or indirectly promote collagen synthesis (47, 48). Furthermore, TAMs secrete factors like IL-10 that induce the expression of monocyte costimulatory molecules and cooperate with regulatory T cells by producing CCL17 and CCL22 to suppress the function of cytotoxic T lymphocytes and participate in the formation of a local immunosuppressive environment (49).

Mononuclear phagocytes and T cells presented distinct quantitative differences between the keloids and the surrounding normal skin tissues. Further dimensionality reduction clustering revealed that T cells were subdivided into six cell types, including naïve T cells, CD4+ effector memory T cells, CD8+ effector T cells, CD8+ mucosal-associated invariant T cells, CD4+ regulatory T cells, and proliferating T cells, of which the proportion of CD8+ effector T cells was predominant in keloids. The results of functional enrichment analysis suggested that T-cell subtypes in keloid and normal tissues were associated with epithelial mesenchymal transition and angiogenesis. The naïve T cells are enriched in homeostatic skin after receiving antigen presentation from Langerhans cells and dendritic cells, coordinated by specific chemokine receptors like CCR4, and gradually start clonal proliferation and cytokine release (50, 51). The decreased ratio of naïve T cells and proliferating T cells and the increased ratio of effector T cells are consistent with the previous knowledge that keloids are closely associated with a regional chronic inflammatory response.

The findings of cellular communication implicated that the macrophages may interact with fibroblasts through TGFB2-TGFBR2, TGFB3-TGFBR3, CCL2-CCR1, and CCL2-CCR5 binding. Previous studies have suggested that in normal tissues, a symbiotic relationship exists between fibroblasts and macrophages in a two-cell circuit, interacting with each other for mutually required growth factor signals. This means that fibroblasts maintain macrophage proliferation by providing CSF1, while fibroblast survival is dependent on PDGF ligands provided by macrophages (52). In fibrotic diseases where fibroblasts are the driver cells, fibroblasts provide signals for macrophage chemotaxis, resident, and activation by maintaining the expression of CSF1, interleukin 6, and the macrophage chemokine CCL2 (52, 53). Activated macrophages secrete multiple factors such as TGF-β, which individually or synergistically drive fibroblast proliferation and activation. Previous studies have reported that activated macrophages are key cells involved in extracellular matrix degradation and collagen breakdown (54); therefore, the dual role and polarization state of macrophages and their interaction with fibroblasts influence the extent of fibrosis.

A keloid is the paradigm of cutaneous fibrosis, and many keloid-based findings have been confirmed in other diseases (2). Pro-fibrotic macrophages have been identified in the single cell profiles of idiopathic pulmonary fibrosis, which upregulate extracellular matrix–related gene expression like SPARC and MMP9 and can construct autocrine feedback loops by expressing CSF1 (55). Aran et al. identified macrophage heterogeneity in a model of lung fibrosis and identified the subpopulations of macrophages that promote mesenchymal formation in the fibrotic fraction and characterized specific markers with therapeutic implications, including CX3CR1 and PDGF-AA (56). In liver fibrosis, macrophages are involved in the liver inflammation, metabolism, and activation of hepatic stellate cells to promote fibrosis and can facilitate the decomposition of the extracellular matrix during the degenerative phase, and clinical trials have shown that the use of autologous macrophage infusion can improve the level of cirrhosis (57, 58). CD163+ macrophages express chemokines associated with perivascular inflammation, and they recruit inflammatory cells into the perivascular environment in scleroderma (59). The heterogeneity and different activation states of macrophages may have different effects on fibrosis in scleroderma (60). This fits well with our findings of macrophage changes in keloid formation and their close association with fibroblasts. Furthermore, studies in recent years have gradually identified mesenchymal fibroblasts as one of the most contributing cells in keloids. This phenomenon may be of general significance, as the expression of the markers POSTN and COMP of mesenchymal fibroblasts has also been observed in scleroderma fibroblasts (9, 61).

In conclusion, our study was based on single-cell sequencing technology to deeply explore the immune cell infiltration in keloid, and we identified differences in the infiltration of macrophages, mast cells, and dendritic cells between normal tissues and keloids, especially the close interaction between macrophages and fibroblasts in keloids, which could, to some extent, contribute to our understanding of the pathogenesis of keloids.

## Data availability statement

The datasets presented in this study can be found in online repositories. The name of the repository and accession number can be found below: NCBI Sequence Read Archive; PRJNA844167.

## Ethics statement

The studies involving human participants were reviewed and approved by the Medical Ethics Committee of Peking Union Medical College Hospital (Beijing, China). The patients/participants provided their written informed consent to participate in this study.

## Author contributions

CF, MS, YX, and YongH performed the data analysis and were major contributors to the preparation of the manuscript. KH, YouW, and ZZ provided technical support in the methodology of the raw letter analysis. CF, KS, TM, HL, ZL, YingW, and YanH made significant contributions to the design of the study. CF, YouW, and YongH confirmed the authenticity of all raw data. All authors read and approved the final manuscript.

## Funding

This study was supported by The National Natural Science Foundation of China (81871538) and Beijing Municipal Commission of Science and Technology (Z191100006619009).

## Acknowledgments

Thanks to Singleron team for the continuous support.

## Conflict of interest

The authors declare that the research was conducted in the absence of any commercial or financial relationships that could be construed as a potential conflict of interest.

## Publisher’s note

All claims expressed in this article are solely those of the authors and do not necessarily represent those of their affiliated organizations, or those of the publisher, the editors and the reviewers. Any product that may be evaluated in this article, or claim that may be made by its manufacturer, is not guaranteed or endorsed by the publisher.

## Supplementary material

Supplementary Figure 1 Differentially expressed genes (DEGs) in cellular subtypes between keloids and normal skin tissues. **(A)** Heatmap illustrating DEGs in fibroblast subtypes. **(B)** Heat map exhibiting DEGs in macrophage subtypes.

Supplementary Figure 2 Spatial cell cluster analysis of keloids scanned by Hyperion imaging mass cytometry. **(A)** Immunohistochemical field for the selected Hyperion tissue imager scan on 5 mm×5 mm formalin-fixed, paraffin-embedded tissue slide (Gross appearance, 40X and 100X). **(B)** Cell cluster analysis the specimen. t-SNE descending dimension map of different cell types. **(C)** Representative images of tissue mass spectrometry stained with CD3, CD4, CD11c, CD45RA, CD68 and other antibodies. The picture on the right is a 5 times magnification of the yellow box on the left.

Supplementary Figure 3 Comparison of cell counts between keloids and normal skin tissues in T-cell subpopulations.

Supplementary Figure 4 Comparative expression of pro- and anti-inflammatory cytokines and chemokines between keloids and normal skin tissues in general **(A)**, fibroblasts **(B)**, macrophages **(C)**, dendritic cells **(D)** and T cells **(E)**.
