# Supplementary material for: Single-cell RNA sequencing reveals distinct immunology profiles in human keloid
Source: Front Immunol. 2022 Aug 3;13:940645. doi: 10.3389/fimmu.2022.940645 (PMC9381754; doi:10.3389/fimmu.2022.940645)
Supplement: Supplementary file 1 [file Table_1.docx]

**Supplementary Table 1. General characteristics of the included keloid patients (12 cases).**

|  | Keloid (n = 12) |
| --- | --- |
| Age (years, mean ± SD) | 35.50 ± 17.84 |
| Male sex (No.) | 3 (25.00%) |
| Family History | No (n = 12, 100.00%) |
| Staying up late | No (n = 12, 100.00%) |
| Smoking | No (n = 12, 100.00%) |
| Drinking | No (n = 12, 100.00%) |
| Chronic dietary |  |
| Preference for spicy food | No (n = 12, 100.00%) |
| Preference for greasy food | Yes (n = 12, 100.00%) |
| Preference for white meat | Yes (n = 12, 100.00%) |
| Occupation |  |
| Retiree | 2 (16.67%) |
| Student | 4 (33.33%) |
| Staff | 2 (16.67%) |
| Unemployed | 3 (25.00%) |
| Others | 1 (8.33%) |

**Supplementary Table 2. Lineage-specific marker genes involved in cell annotation after hierarchical clustering.**

| Abbreviation | Cell type | Marker |
| --- | --- | --- |
| General cellular annotation | | |
| TCells | T cells | CD2, CD3D, TRAC, TRBC2 |
| LangerhansCells | Langerhans cells | CD207, CD1A |
| MPs | Mononuclear phagocytes | CD14, CD1C, VCAN, C1QA, FCER3A |
| MastCells | Mast cells | TPSAB1, TPSB2, CPA3 |
| Fibroblasts | Fibroblasts | DCN, COL1A1, COL1A2 |
| ECs | Endothelial cells | PECAM1, CDH5, VWF, KDR |
| Melanocytes | Melanocytes | MLANA, PMEL, DCT, TYRP1 |
| SGCs | Sweat gland cells | DCD, AQP5 |
| MuralCells | Mural cells | RGS5, ACTA2, TAGLN, MYLK |
| Keratinocytes | Keratinocytes | KRT1, KRT10, KRT14, KRT5 |
| Schwann cells | Schwann cells | S100B, PMP22, PLP1 |
| Fibroblast subtype annotation | | |
| MF | Mesenchymal fibroblasts | ASPN, POSTN, COMP |
| SPF | Secretory papillary fibroblasts | APCDD1, COL18A1, COL13A1 |
| PF | Pro-inflammatory fibroblasts | APOE, CCL19, CXCL3 |
| SRF | Secretory reticular fibroblasts | WISP2, ANGPTL1, MFAP5 |
| MPs subtype annotation |  |  |
| Macrophages | Macrophages | C1QA, MRC1, APOE, CD68 |
| cDC1 | Conventional type 1 dendritic cells | XCR1, CLEC9A, THBD |
| cDC2 | Conventional type 2 dendritic cells | CD1C, CD1E, FCER1A |
| MatureDCs | Mature dendritic cells | LYZ, LAMP3, CCR7 |
| ProliferatingMPs | Proliferating mononuclear phagocytes | MKI67, TOP2A, CD1C, C1QA |
| pDC | Plasmacytoid dendritic cells | LYZ, LILRA4, IRF4, GZMB, JCH, AIN |
| MastCells | Mast cells | TPSAB1, TPSB2, CPA3 |
| Neutrophils | Neutrophils | CSF3R, CXCR2, FCGR3B |
| Monocytes | Monocytes | CD14, VCAN, FCN1 |
| T cells subtype annotation | | |
| CD4NaiveT | CD4+ Naïve T cells | CD3D, TCF7, IL7R |
| CD4Tem | CD4+ effector memory T cells | CD4, DUSP4, CXCR6 |
| CD8Teff | CD8+ effector T cells | CD8A, GNLY, NKG7, GZMA, XCL1/2 |
| CD8MAIT | CD8+ mucosal-associated invariant T cells | CD8A, KLRB1, NCR3 |
| Treg | CD4+ regulatory T cells | CD3D, FOXP3, IL2RA, CTLA4, IKZF2 |
| ProliferatingTCells | Proliferating T cells | CD3D, TOP2A, MKI67 |

**Supplementary Table 3. The absolute cell numbers of different cell types in each sample by single cell RNA sequencing**

|  |  |  |  | Cell Types | |  |  |  |  |  |  |
| --- | --- | --- | --- | --- | --- | --- | --- | --- | --- | --- | --- |
| Samples | ECs | Fibroblasts | Keratinocytes | Langerhans | Macrophages | Mast | Melanocytes | Mural | SGCs | Schwann | TCells |
| K1 | 3746 | 5443 | 12166 | 166 | 957 | 1807 | 90 | 2321 | 25 | 258 | 984 |
| K2 | 1459 | 94 | 12772 | 142 | 88 | 160 | 31 | 544 | 12 | 5 | 265 |
| K3 | 3269 | 954 | 12970 | 254 | 278 | 398 | 96 | 1393 | 3828 | 39 | 467 |
| K4 | 617 | 166 | 13267 | 11 | 2 | 10 | 137 | 450 | 113 | 34 | 28 |
| K5 | 651 | 131 | 13555 | 23 | 20 | 26 | 190 | 362 | 1161 | 9 | 34 |
| K6 | 206 | 50 | 13320 | 0 | 4 | 76 | 256 | 135 | 231 | 24 | 29 |
| K7 | 170 | 136 | 15546 | 43 | 19 | 80 | 233 | 101 | 51 | 12 | 47 |
| K8 | 1282 | 219 | 9149 | 109 | 81 | 277 | 91 | 382 | 97 | 27 | 223 |
| K9 | 1624 | 302 | 11983 | 141 | 195 | 567 | 644 | 778 | 174 | 107 | 759 |
| K10 | 562 | 57 | 9418 | 78 | 39 | 81 | 520 | 190 | 1164 | 17 | 231 |
| K11 | 532 | 42 | 15488 | 114 | 26 | 103 | 135 | 109 | 1658 | 7 | 43 |
| K12 | 4866 | 6346 | 1766 | 302 | 1390 | 4402 | 140 | 2228 | 150 | 1301 | 1142 |
| N1 | 82 | 18 | 8612 | 77 | 31 | 18 | 27 | 48 | 325 | 1 | 115 |
| N2 | 214 | 86 | 17489 | 130 | 72 | 84 | 100 | 129 | 990 | 1 | 120 |
| N3 | 462 | 278 | 29986 | 106 | 162 | 128 | 128 | 303 | 4401 | 1 | 199 |
| N4 | 123 | 47 | 10092 | 2 | 18 | 12 | 183 | 65 | 1429 | 0 | 31 |
| N5 | 112 | 105 | 11496 | 3 | 8 | 23 | 50 | 92 | 2275 | 2 | 55 |
| N6 | 32 | 14 | 16062 | 2 | 0 | 12 | 171 | 6 | 200 | 0 | 51 |
| N7 | 188 | 29 | 12640 | 8 | 13 | 15 | 202 | 47 | 929 | 0 | 68 |
| N8 | 400 | 175 | 9216 | 15 | 186 | 91 | 149 | 146 | 3371 | 3 | 235 |
| N9 | 8 | 9 | 21682 | 1 | 4 | 35 | 37 | 18 | 675 | 0 | 123 |
| N10 | 209 | 77 | 10065 | 27 | 34 | 32 | 317 | 160 | 2765 | 0 | 128 |
| N11 | 246 | 76 | 16266 | 63 | 22 | 14 | 200 | 156 | 2196 | 1 | 52 |
| N12 | 3124 | 4107 | 1120 | 308 | 1411 | 1571 | 178 | 2997 | 5886 | 107 | 1737 |

**Supplementary Table 4. The average cell count and cell proportion of different cell types in the samples.**

| Cell Type | Cell count | | Cell proportion | |
| --- | --- | --- | --- | --- |
|  | (Median, interquartile range） | | (%) | |
|  | Keloid | Normal | Keloid | Normal |
| ECs | 966.50 (554.50, 2035.25) | 198.50 (104.50, 284.50) | 8.91% | 2.43% |
| Fibroblasts | 151.00 (84.75, 465.00) | 76.50 (26.25, 122.50) | 6.54% | 2.35% |
| Keratinocytes | 12871.00 (11341.75, 13378.75) | 12068.00 (9852.75, 16571.75) | 66.35% | 76.95% |
| LangerhansCells | 111.50 (38.00, 148.00) | 21.00 (2.75, 84.25) | 0.65% | 0.35% |
| Macrophages | 60.00 (19.75, 215.75) | 26.50 (11.75, 94.50) | 1.45% | 0.92% |
| MastCells | 131.50 (79.00, 440.25) | 27.50 (14.75, 85.75) | 3.75% | 0.95% |
| Melanocytes | 138.50 (94.75, 238.75) | 160.00 (87.50, 187.25) | 1.20% | 0.81% |
| MuralCells | 416.00 (176.25, 931.75) | 110.50 (47.75, 157.00) | 4.22% | 1.95% |
| SGCs | 162.00 (85.50, 1161.75) | 1812.50 (865.50, 2916.50) | 4.07% | 11.89% |
| SchwannCells | 25.50 (11.25, 56.00) | 1.00 (0, 1.25) | 0.86% | 0.05% |
| TCells | 227.00 (40.75, 540.00) | 117.50 (54.25, 145.75) | 2.00% | 1.36% |
