# Supplementary figures and images for: Single-cell RNA sequencing reveals distinct immunology profiles in human keloid
Source: Front Immunol. 2022 Aug 3;13:940645. doi: 10.3389/fimmu.2022.940645 (PMC9381754; doi:10.3389/fimmu.2022.940645)

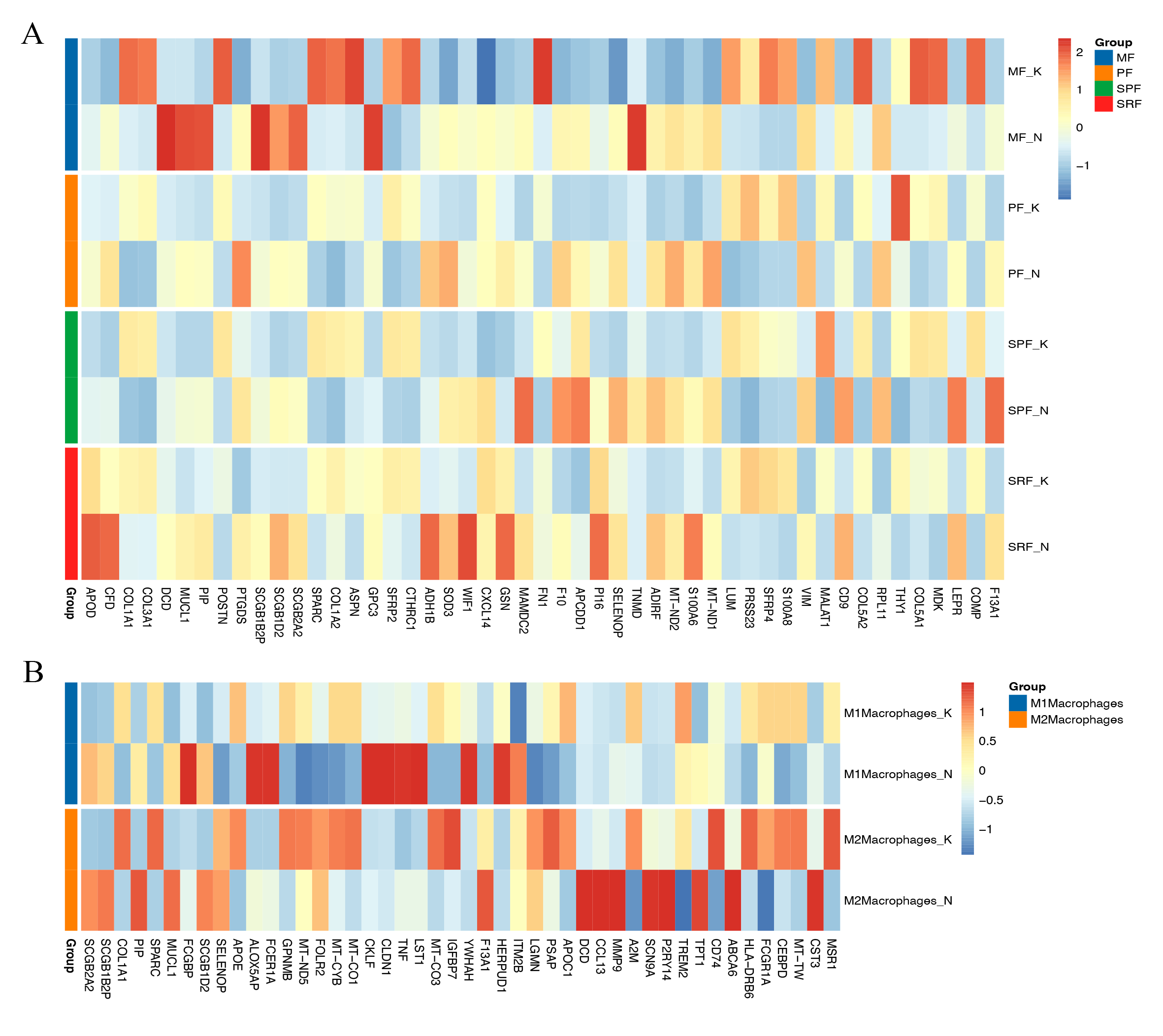

Supplement: Supplementary Figure 1 — Differentially expressed genes (DEGs) in cellular subtypes between keloids and normal skin tissues. (A) Heatmap illustrating DEGs in fibroblast subtypes. (B) Heat map exhibiting DEGs in macrophage subtypes. [file Image_1.tif]

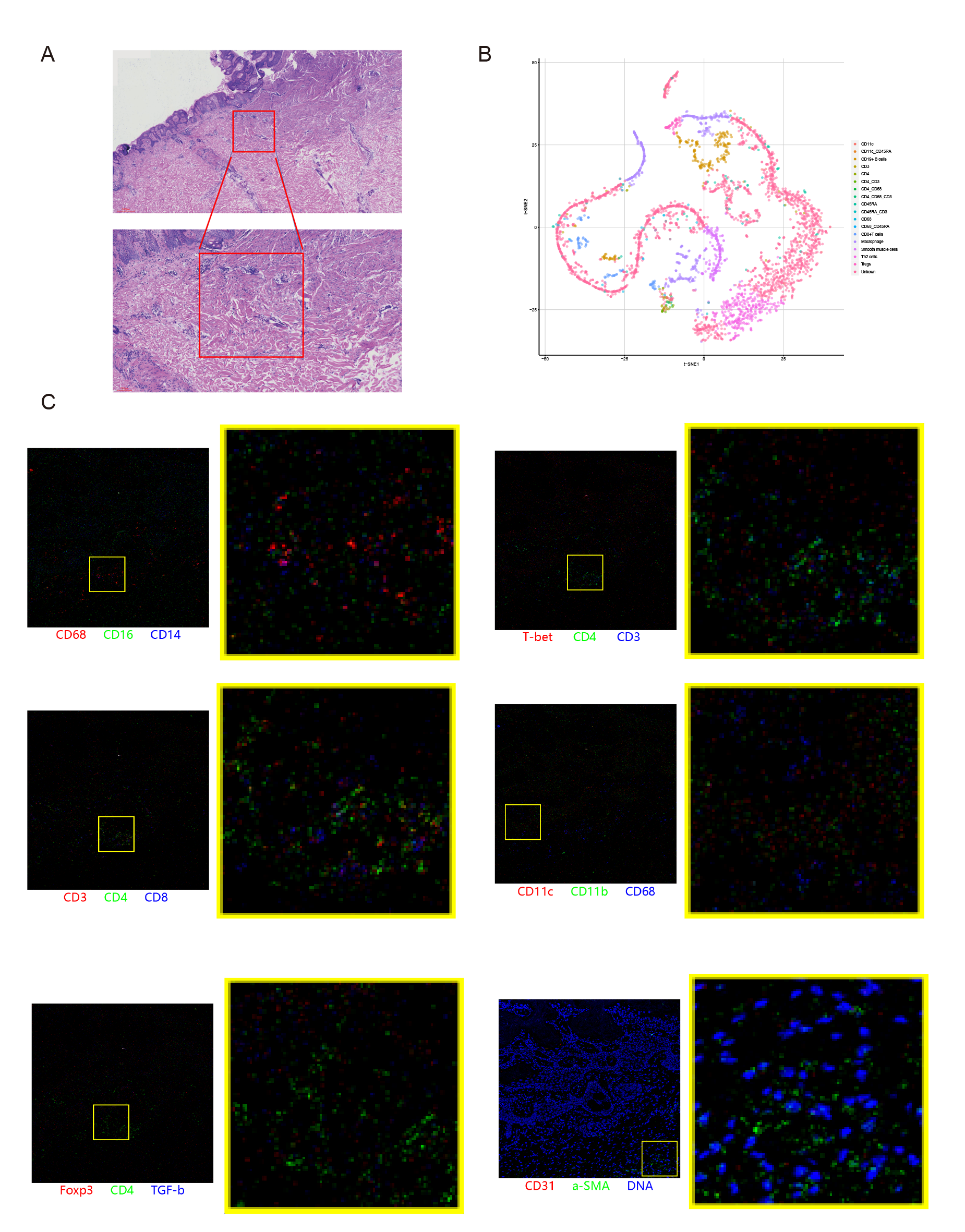

Supplement: Supplementary Figure 2 — Spatial cell cluster analysis of keloids scanned by Hyperion imaging mass cytometry. (A) Immunohistochemical field for the selected Hyperion tissue imager scan on 5 mm×5 mm formalin-fixed, paraffin-embedded tissue slide (Gross appearance, 40X and 100X). (B) Cell cluster analysis the specimen. t-SNE descending dimension map of different cell types. (C) Representative images of tissue mass spectrometry stained with CD3, CD4, CD11c, CD45RA, CD68 and other antibodies. The picture on the right is a 5 times magnification of the yellow box on the left. [file Image_2.tif]

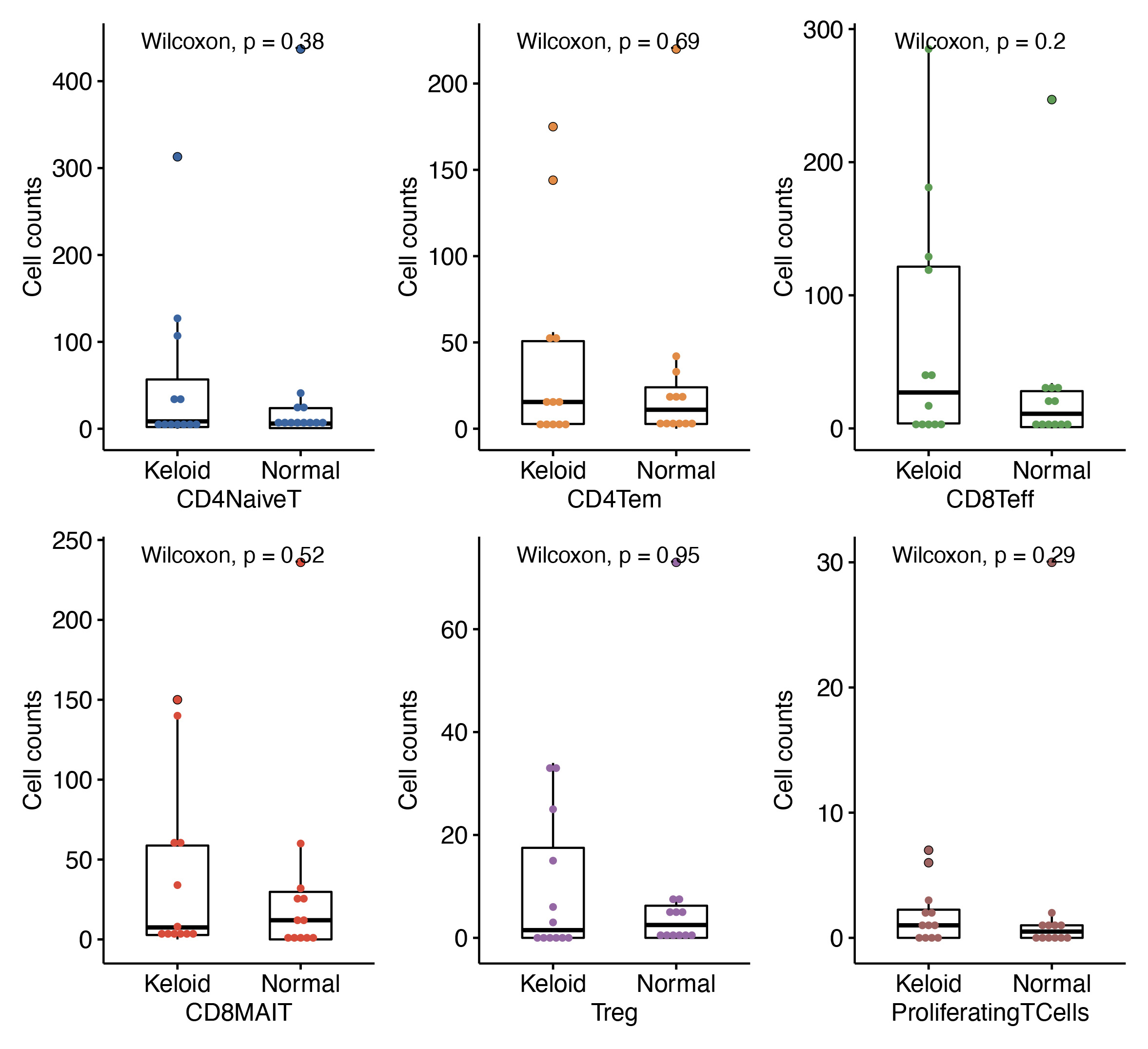

Supplement: Supplementary Figure 3 — Comparison of cell counts between keloids and normal skin tissues in T-cell subpopulations. [file Image_3.jpeg]
